# Predictive Models for *Tomato Spotted Wilt Virus* Spread Dynamics, Considering *Frankliniella occidentalis* Specific Life Processes as Influenced by the Virus

**DOI:** 10.1371/journal.pone.0154533

**Published:** 2016-05-09

**Authors:** Pamella Akoth Ogada, Dany Pascal Moualeu, Hans-Michael Poehling

**Affiliations:** 1 Institute of Horticultural Production Systems, Department of Phytomedicine, Gottfried Wilhelm Leibniz Universität Hannover, Herrenhäuser Str. 2, 30419, Hannover, Germany; 2 Institute of Horticultural Production Systems, Department of Vegetable Systems Modelling, Gottfried Wilhelm Leibniz Universität Hannover, Herrenhäuser Str. 2, 30419, Hannover, Germany; Washington State University, UNITED STATES

## Abstract

Several models have been studied on predictive epidemics of arthropod vectored plant viruses in an attempt to bring understanding to the complex but specific relationship between the three cornered pathosystem (virus, vector and host plant), as well as their interactions with the environment. A large body of studies mainly focuses on weather based models as management tool for monitoring pests and diseases, with very few incorporating the contribution of vector’s life processes in the disease dynamics, which is an essential aspect when mitigating virus incidences in a crop stand. In this study, we hypothesized that the multiplication and spread of *tomato spotted wilt virus* (TSWV) in a crop stand is strongly related to its influences on *Frankliniella occidentalis* preferential behavior and life expectancy. Model dynamics of important aspects in disease development within TSWV-*F*. *occidentalis*-host plant interactions were developed, focusing on *F*. *occidentalis*’ life processes as influenced by TSWV. The results show that the influence of TSWV on *F*. *occidentalis* preferential behaviour leads to an estimated increase in relative acquisition rate of the virus, and up to 33% increase in transmission rate to healthy plants. Also, increased life expectancy; which relates to improved fitness, is dependent on the virus induced preferential behaviour, consequently promoting multiplication and spread of the virus in a crop stand. The development of vector–based models could further help in elucidating the role of tri-trophic interactions in agricultural disease systems. Use of the model to examine the components of the disease process could also boost our understanding on how specific epidemiological characteristics interact to cause diseases in crops. With this level of understanding we can efficiently develop more precise control strategies for the virus and the vector.

## Introduction

*Tomato spotted wilt virus* (TSWV) belongs to the genus *Tospovirus*, the only plant infecting genus in the family *Bunyaviridae* [1][2]. TSWV is transmitted exclusively by thrips, with *Frankliniella occidentalis* (Western flower thrips) being the most efficient vector due to its equally large host range [1][3][4]. *Tospoviruses* and their complex virus–vector interaction were ranked second in a recent survey by plant virologists based on the perceived economic and scientific importance [5].

Transmission of TSWV by *F*. *occidentalis* occurs in a propagative and persistent manner [1][6]. Only adults (and partly the second instars) can transmit the virus, but only when the virus is acquired at the early larval stage of the thrips’ life cycle [7][8][9][10][11], and after the virus has replicated and circulated inside the host midgut [12][13]. An increase in the viral protein in the thrips vector during their development is an indication of TSWV replication in the host [7][14][3][11]. TSWV initially infects the midgut, replicating first in the epithelium and then in the muscle fibres surrounding the foregut and the midgut of the developing larvae. The virus then spreads to the salivary glands where it also replicates to achieve a high virus titer in the saliva and from which it is then transmitted to the plants during feeding [7][14][15][16][17][18]. TSWV acquisition by second larval stage or by adults support infections in the midgut tissues, but the virus does not spread to the salivary glands therefore cannot be transmitted to plants [19][20][21].

Virus replication in the tissues of its thrips host suggests the likelihood for pathological effects that could affect the fitness and performance of the vector. However, consistent demonstration of such effects has proven difficult due to the complex virus-vector-host plant interaction [22]. Moreover, studies are now available showing varying effects between vector and pathogen on development rate, survival, reproduction rate and preferential behaviour of the vector [23][24][25][26][27][28][29], which can further influence the transmission and spread of the virus. However, it has proven difficult to predict these observed influences in vector and plant populations due to the complexity of *Tospovirus*-vector-host plant interaction. Mathematical models can therefore help to quantify the virus propagation dynamics in a crop stand as influenced by virus induced vector life’s processes.

Nevertheless, despite both TSWV and *F*. *occidentalis* being great threats to the global economy, very little effort has been made to come up with a wholesome model specifically forecasting TSWV epidemiology considering the complex and mutualistic interactions in the triangle virus-vector-host plant. According to a review paper by Jones et al [30], by the year 2010 there were only 2 published papers on general predictive models for TSWV epidemics involving thrips vector [31][32]. These papers together with several modelling studies that followed, mainly focused on the importance of weather conditions in the mentioned interaction [33][34], with only a few incorporating the role of vectors in varying ways. For example, influence of weather factors on vector’s population dynamics [35][36], vector abundance [37], vector activities [38] and vector species diversity [39]. Initial work on developing models from an arthropod-centred perspective rather than the usual plant-centred perspective has been reported [40]. Most recently, Jeger et al. [41] published a first study which focused on the contribution of thrips based parameters such as thrips age at virus acquisition and transmission, incorporated into a general predictive model of transmission characteristics within TSWV-thrips interaction.

We therefore presume that the application of a more specific vector–centred model (*F*. *occidentalis*-TSWV) incorporating virus induced vector life processes influencing the disease epidemic development, would provide better understanding into the often aggressive spread of TSWV in crop stands, and also allow more precise forecasting for efficient management strategies for both *F*. *occidentalis* and TSWV.

## Methodology

### Study system

Several examples of behavioural changes in herbivores serving as vectors as a result of hosting (or infection by) the relevant pathogens have been reported [42][43][44][45][46][47]. Most often such changes favour the replication and/or spread of the pathogen. For instance, TSWV benefits by modifying the behaviour and fitness, as well as improving the host plant quality for its vector [24][42]. Other studies have related the improved fitness of the viruliferous (virus carrying) thrips to triggered immunity [48], as the insect lack an adaptive immune system [49]. Together with the virus induced preferential behaviour, these ensure longer survival of the infected vector [24] and thus promoting multiplication and spread of the virus. Which indicates a mutualistic relationship. Furthermore, exposure of *F*. *occidentalis* to TSWV has been reported to cause significant reduction in development time, which has been alleged to be due to biochemical reactions within the vector as a result of the replicating virus [24][42]. These reported viral influences on the vector are independent of the age at acquisition, the transmission potential of the vector, or the virus isolate [19][24][20]. Several studies have analysed the characteristics of these epidemiological consequences of persistent-propagative transmission [50][51]. However, they do not consider the specific virus manipulated life processes on the vector, as well as the lack of trans-ovarian (vertical) transmission of TSWV; meaning that each generation must re-acquire the virus for the disease epidemic to continue [7][3][11]. Even though larvae 2 (L2) and the adults can ingest TSWV particles, they are not able to transmit the virus due to the role of a tissue barrier from the midgut muscle cells to salivary glands [15][52][9][20]. This interdependency between vector life-stage and virus transmission involves multiple infection and dissemination barriers related to the developmental changes (life cycle) of thrips vectors [7][53][54].

This paper aims to provide a specific analysis of TSWV disease dynamics in a model framework consisting of specific transmission characteristics in the *Tospovirus*-thrips interaction, focusing on the influences of the TSWV on *F*. *occidentalis* biology in terms of life expectancy (survival) and preferential behaviour.

For model development assumptions, we refer to the general approaches reported in most of the plant viruses’ predictive models [50][40][41][38], with additional transmission characteristics focusing on the *F*. *occidentalis* developmental as well as behavioural aspects as influenced by TSWV [24][42].

### Model description and development

The influence of TSWV on *F*. *occidentalis*’ preferential behaviour patterns, development time, survival, as well as the key aspects of the vector lifecycle such as developmental stages, forms the basis of the hereby reported model. We consider two scenarios for the host plant; TSWV presence (infected, I) or absence (healthy, H). The basis of the assumptions is that TSWV presence influences the transmission intensity of *F*. *occidentalis* by its influence on adults’ preferential behaviour and survival, which further affects the disease incidence in a crop stand.

*F*. *occidentalis* adults status can be described as **Healthy** (***A***_***N***_); if it is not exposed to TSWV infected plant by feeding, **Infected** (***A***_***I***_); if the vector fed on infected host plant but at a less crucial stage for acquisition in view of transmission, that is the L2 (***L*2**_***N***_) and/or the adult stage (***A***_***N***_), or **Transmitter** (**A**_**T**_); if the vector fed on infected host plants at the Larvae 1 (L1) stage which is essential for later transmission by adults. **A**_**T**_ is the only group of adults that are able to transmit the virus.

Due to the high numbers of *F*. *occidentalis* classes and the interactions between them, we chose to use a deterministic model consisting of differential equation systems.

The model describing the disease and the vector dynamics was developed based on the following assumptions;

- Virus transmission to host plants is dependent on the presence of transmitter vectors and healthy host plant.
- Acquisition of the virus by the vector is by feeding on an infected host plant.
- The feeding stages of the vector are L1, L2 and adult; pre-pupa and pupa stages do not feed.
- Only L1 that emerge on infected host plants and acquire the virus can become transmitters as adults.
- L2 and adults are able to acquire the virus and become infected but not transmitters.
- Larval stages (L1 and L2) are mobile but restricted to the plant where they hatched from eggs due to lack of wings.
- Pre-pupae and pupae stages are immobile, do not feed and spend their entire time in the growth media i.e. not on the plant.
- The virus replicates inside the vector and is transmitted transstadially.
- Adults are the only stage that can move from plant to plant, therefore, essential for the virus transmission and thus spread.
- Transovarial transmission of the virus is not possible.
- Exposure of the vector to the virus leads to improved fitness, only when feeding on healthy host plant after the exposure.
- Preferential behaviour of the adults to the host plant is influenced by their status i.e. viruliferous adults (infected or transmitters) prefer healthy host plants, while the virus-free (healthy) prefer infected host plants.

### Parameter estimation

Parameter estimation was based on recent findings which indicated a form of mutualism between TSWV and its vector *F*. *occidentalis* [24][42]. Development and behaviour of *F*. *occidentalis* (exposed or non-exposed to TSWV) on host plants with different health status (+/- TSWV) is reported to be different. As TSWV infection on the host plants influences the vector biology by reducing the development time, increasing survival and changing the preferential behaviour of the vector, only if the virus exposed vectors were later reared on healthy host plant as adults [42][24]. Therefore, the life cycle of the vector will vary accordingly (Table 1). All compartments of the models are summarized in Table 2 with their corresponding descriptions. Initial values of the vector population and virus abundance were estimated (Table 2) to evaluate the interaction.

**Table 1:** Development time values of different life stages of *F*. *occidentalis* growing on host plants with different TSWV status (infected or healthy host plants).

Developmental stages and time of *F*. *occidentalis* on healthy host plants [55][56]		Development time on TSWV infected host plants [24]
Egg→L1 (Emergence)	2–4 days	2-3days
L1→L2	1–2 days	1day
L2→Pre-pupa	2–4 days	2days
Pre-pupa→Pupa	1–2 days	1day
Pupa→Adult	1–3 days	1-2days
Egg→Adult	14-21days	9-10days
Adult lifetime	30–45 days	42-51days

**Table 2:** List of model compartments, symbols and their descriptions, with hypothetical initial values.

Symbol	Description	Initial values
H	Fraction of healthy plants	791/800
I	Fraction of infected plants	9/800
E_H_	Amount of eggs in healthy plants	6000
E_T_	Amount of eggs in infected plants	108
L1_H_	Amount of L1 in healthy plants	5760
L1_T_	Amount of L1 in infected plants	99
L2_H_	Amount of L2 in healthy plants	3000
L2_I_	Amount of L2 emerging in newly infected plants	0
L2_T_	Amount of L2 in infected plants	45
PP_N_	Amount of non-infected pre-pupae from healthy plants	0
PP_I_	Amount of infected pre-pupae from infected plants	0
PP_T_	Amount of pre-pupae from infected plants that will become transmitters	0
P_N_	Amount of non-infected pupae from healthy plants	0
P_I_	Amount of infected pupae from infected plants	0
P_T_	Amount of transmitter pupae from infected plants	0
A_N_	Amount of non-infected *F*. *occidentalis* adults	1200
A_I_	Amount of infected *F*. *occidentalis* adults	310
A_T_	Amount of transmitter *F*. *occidentalis* adults	600

### Model dynamics

**Fig 1:**
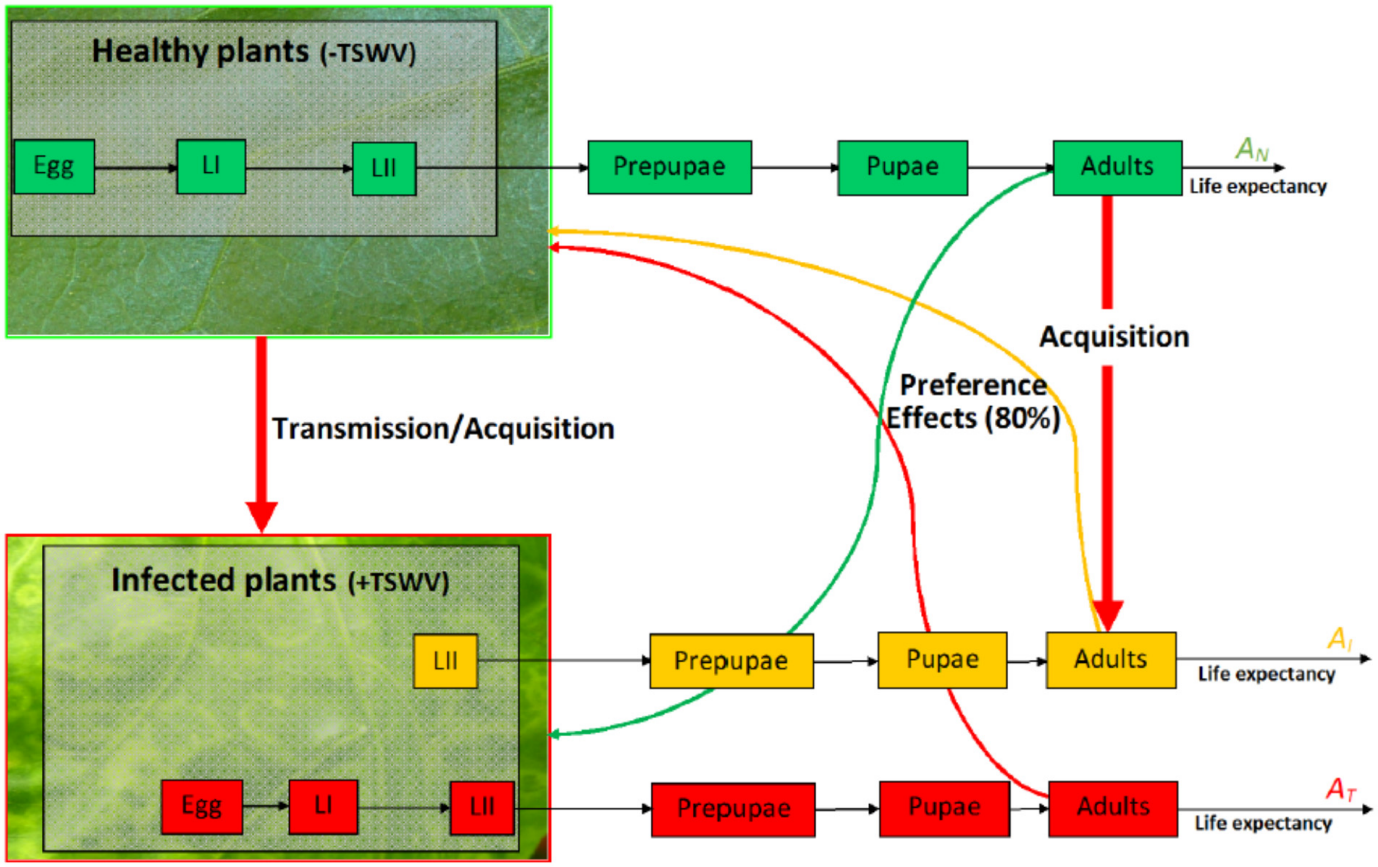
Flowchart describing TSWV–*F*. *occidentalis* interaction dynamics. The three coloured pathways represent exposure status of *F*. *occidentalis* to TSWV; Green → Healthy, Orange → Infected and Red → Transmitter individuals. The green, yellow, and red arrows indicate the virus induced preferential behaviour of adults, with a preferential factor of 80%.

A flowchart illustration of selected influences of TSWV on *F*. *occidentalis* is given below (Fig 1), indicating all developmental classes of the model. A comparison is made between *F*. *occidentalis* that fed on virus infected (+TSWV) and on healthy (-TSWV) host plants, in terms of TSWV influences on their life expectancy as well as preferential behaviour. There are three groups of adults as a result of the exposure status; Healthy, Transmitters and Infected adults, already described above.

### Description of developmental classes dynamics

#### Eggs

Eggs are either laid on TSWV infected or healthy host plants based on the preferential behaviour of the adults, which is influenced by their infection status. Since transovarial (vertical) transmission is not possible, all eggs are considered healthy, even if they are laid on infected host plant. The assumption of preferential behaviour is based on our recent study with *F*. *occidentalis* and TSWV [42]. The amount of eggs laid on healthy plants is determined by the number of adults in each category and their infection status, and is modelled by:

$$\mu \text{(}p_{IH}\;*\;A_{I}\text{ + }p_{NH}\;*\;A_{N}\text{) }*\;H$$

where ***p***_***IH***_ = **0.8** and ***p***_***NH***_ = **0.2,** are preferential factors effects on the infected and non-infected adult *F*. *occidentalis* to feed on healthy plants. *μ* is the average number of eggs laid per adult per day. The total amount of eggs laid on infected plants per day is given by:

$$\mu ((1-p_{IH})\;*\;A_{I}+(1-p_{NH})*A_{N}+(1-p_{TH})*A_{T})*I$$

When transmitter adults lay eggs on healthy plants, we assume that they would feed on the plant transmitting the virus, and thus changing the status of the plant. The complete dynamics of eggs is given below.

In healthy plants:

$$\frac{dE_{H}}{dt}\;=\;\mu (p_{IH}A_{I}\;+\;p_{NH}A_{N})H-\lambda_{T}HE_{H}-(\eta_{EH}\;+\;\tau_{EH})E_{H}$$ (1)

In infected plants:

$$\frac{dE_{T}}{dt}=\mu ((1-p_{IH})A_{I}\;+\;(1-p_{NH})A_{N}\;+\;(1-p_{IH})A_{T})I+\lambda_{T}HE_{H}-(\eta_{ET}+\tau_{ET})E_{T}$$ (2)

where, $\frac{\text{1}}{\eta_{i}},\;i\text{ = }EH,\;ET$ is the average number of days required for eggs to emerge in healthy and infected plants (3 days and 2.5 days) respectively. ***τ***_***i***_ **= 20**%, i = EH,EV is the estimated fraction of eggs destroyed per day.

The parameter ***λ***_***T***_ which models the transmission rate of the infection to plants is defined by:

$$\lambda_{T}=p_{TH}\beta_{I}\frac{A_{T}}{A_{T}\text{ + }A_{I}\text{ + }A_{N}}$$ (3)

*ß*_I_ is the contact rate that leads to infection and ***p***_***TH***_ = **0.8**, is the preferential factor of the transmitter adult *F*. *occidentalis* to feed on healthy plants.

#### Larvae 1 (L1)

L1 emerging from eggs laid on the infected plants will automatically feed on the infected plants, and since this is a crucial stage for acquisition in view of transmission by adults, these L1 are referred to as transmitters. While the L1 that emerged from the healthy host plants will feed on the healthy plants and thus are referred to as healthy. However, in case of eggs laid by the transmitter adults on the healthy plants as a result of the preferential behaviour, there is a possibility of TSWV transmission to the healthy plants during egg laying, assuming that they fed on the plants, therefore changing the status of healthy plants to TSWV infected, and thus, the L1 emerging and feeding on these newly infected host plants also become transmitters. The dynamics of this population is given by;

In healthy plants:

$$\frac{dL1_{H}}{dt}=\eta_{EH}E_{H}-\lambda_{T}H\;L1_{H}-(\eta_{L1H}+\tau_{L1H})L1_{H}$$ (4)

In infected plants:

$$\frac{dL1_{T}}{dt}=\eta_{ET}E_{T}+\lambda_{T}H\;L1_{H}-(\eta_{L1T}+\tau_{L1T})L1_{T}$$ (5)

where,$\frac{1}{\eta_{i}},\;i=L1H,\;L1T$ is the average number of days required for L1 to develop to L2 in healthy and infected plants respectively (1.5 days, 1 day). ***τ***_**L1H**_ **= 20%** and ***τ***_**L1T**_ **= 10**% are estimated daily death rates of L1.

#### Larvae 2 (L2)

The transmitter L1 develop to transmitter L2 on the TSWV infected host plants, and the healthy L1 to healthy L2 on the healthy host plants. However, in case of transmission of TSWV to the healthy plants by the transmitter adults, the resulting L2 will feed on the now TSWV infected plants, acquire the virus, and become infected. Nevertheless, transmission by adults resulting from this group is not possible. Some literatures suggest that the early stages of L2 can successfully acquire the virus for transmission at adult stage; however, this has not been adequately validated.

The dynamics of this population is given by;

In healthy plants:

$$\frac{dL2_{H}}{dt}=\eta_{L1H}L1_{H}-\lambda_{T}H\;L2_{H}-(\eta_{L2H}+\tau_{L2H})L2_{H}$$ (6)

In infected plants:

$$\frac{dL2_{I}}{dt}=\lambda_{T}H\;L2_{H}-\text{(}\eta_{L2I}\;+\;\tau_{L2I}\text{)}L2_{I}$$ (7)

$$\frac{dL2_{T}}{dt}=\eta_{L2T}L1_{T}-(\eta_{L2T}\;+\;\tau_{L2T})L2_{T}$$ (8)

where, $\frac{1}{\eta_{i}},\;i=L2H,\;L2I,\;L2T$ are average numbers of days required for L2 to develop to prepupae in healthy and infected plants (3 days, 2 days). ***τ***_***L*2*H***_ **= 20**%, ***τ***_***L*2*I***_ **= 10**% and ***τ***_***L*2*T***_ **= 10**% are estimated daily death rates of L2.

#### Prepupae and pupae

Since these two stages are immobile, do not feed and are primarily in the growth media, i.e. outside the plant, their status is solely dependent on the status of the preceding L2. That is, healthy, infected and transmitter L2 will result to similar categories for both pupal stages. We subdivided each of these classes into three subclasses depending on their status.

The prepupa population dynamics is given as:

$$\frac{dPP_{N}}{dt}=\eta_{L2H}L2_{H}-(\eta_{PPN}\;+\;\tau_{PPN})PP_{N}$$ (9)

$$\frac{dPP_{I}}{dt}=\eta_{L2I}L2_{I}-(\eta_{PPI}\;+\;\tau_{PPI})PP_{I}$$ (10)

$$\frac{dPP_{T}}{dt}=\eta_{L2T}L2_{T}-(\eta_{PPT}\;+\;\tau_{PPT})PP_{T}$$ (11)

The pupa population dynamics is given as:

$$\frac{dP_{N}}{dt}=\eta_{PPN}PP_{N}-(\eta_{PN}+\tau_{PN})P_{N}$$ (12)

$$\frac{dP_{I}}{dt}=\eta_{PPI}PP_{I}-(\eta_{PI}+\tau_{PI})P_{I}$$ (13)

$$\frac{dP_{T}}{dt}=\eta_{PPT}PP_{T}-(\eta_{PT}+\tau_{PT})P_{T}$$ (14)

where, $\frac{1}{\eta_{i}},\;\;i\;=\;PPN,\;PPI,\;PPT;\;PN,\;PI,\;PT$ are the average number of days required for development from prepupa to pupa (1.5 days, 1 day and 1 day); and from pupa to adult, for the healthy, infected and transmitter status respectively (2 days, 1.5 days and 1.5 days). ***τ***_***PPN***_ **= *τ***_***PN***_ **= 20**% and ***τ***_***PPI***_ **= *τ***_***PPT***_ **= *τ***_***PI***_ **= *τ***_***PT***_ **= 10**% are estimated daily death rates of pupae and prepupae.

#### Adults

Adults are the most important stage in the transmission cycle, as they are mobile and can fly from one plant to the other, therefore, are responsible for the spread of TSWV in a crop stand. The status of the adults is also dependent on the pupae status, which results into the three adults’ categories; Healthy, Infected and Transmitters. Unlike the pupal stages, the adults are mobile and feed. Healthy adults can feed on TSWV infected host plants and acquire the virus, which leads to a change in their status from healthy to infected adults, but not transmitters due to the developmental barriers already mentioned. Furthermore, the influence of TSWV on the preferential behaviour can be seen clearly in this stage, due to their ability to fly. And as we already reported, the viruliferous adults; infected and transmitters, show preference towards healthy over the TSWV infected host plants, while the virus free adults prefer TSWV infected over healthy host plants [42][24]. These consequently influence the amount of eggs laid on either infected or healthy host plants, and thus the status of the resulting *F*. *occidentalis* adults in the population, which further influence the spread of TSWV.

The adults’ population dynamics is given as follows:

$$\frac{dA_{N}}{dt}=\eta_{PN}P_{N}-\beta_{A}IA_{N}-\tau_{AN}A_{N}$$ (15)

$$\frac{dA_{I}}{dt}=\eta_{PI}P_{I}+\beta_{A}IA_{N}-\tau_{AI}A_{I}$$ (16)

$$\frac{dA_{T}}{dt}=\eta_{PT}P_{T}-\tau_{AT}A_{T}$$ (17)

where, $\frac{1}{\tau_{i}},\;\;i\;=\;AN,\;AT\text{ and}\;AI$ are adults’ life expectancy depending on the fraction of healthy plants available, and is modelled as:

$$\frac{1}{\tau_{AN}}\;=\;37.5,\;\frac{1}{\tau_{AT}}\;=\;\frac{1}{\tau_{AI}}\;=\;37.5\;+\;10.5(2H\;-\;1)$$

where, H is the fraction of healthy plants. This assumption is based on the fact that life expectancy of adults exposed to TSWV is higher on healthy plants than on infected plants.

Virus acquisition by healthy *F*. *occidentalis* adults is modelled as;

$$(1-p_{NH})\beta_{A}I\;A_{N},$$

where, (1 − *p*_*NH*_) is the preference towards infected plants, and *ß*_A_ is the contact rate between the insect and infected plant.

### Simulations

Numerical simulations of the model were performed using the modeling software Vensim PLE (**Ventana Systems, Inc.,** Harvard, USA) with a Runge-Kutta 4 scheme. Different scenarios were compared to evaluate the impact of TSWV influence on preferential behaviour and life expectancy of *F*. *occidentalis*, on the propagation and spread dynamics of the virus in a plant population. The maximum evaluation time was set at 100 days (after TSWV introduction in a crop stand) and the step sizes were fixed to minimum (0.0078125 days) to avoid any stiffness of the scheme.

## Results

### A. Influence of TSWV induced preferential behaviour of *F*. *occidentalis* on the TSWV-vector-host plant interaction dynamics

The considered dynamics for the interaction were based on reports that exposure of *F*. *occidentalis* to TSWV leads to a change in their preferential behaviour. Non-exposed *F*. *occidentalis* (healthy) prefer TSWV infected host plants; while the TSWV exposed ones significantly prefer healthy host plants.

#### 1. Disease spread dynamics (Acquisition/ Transmission)

**Fig 2:**
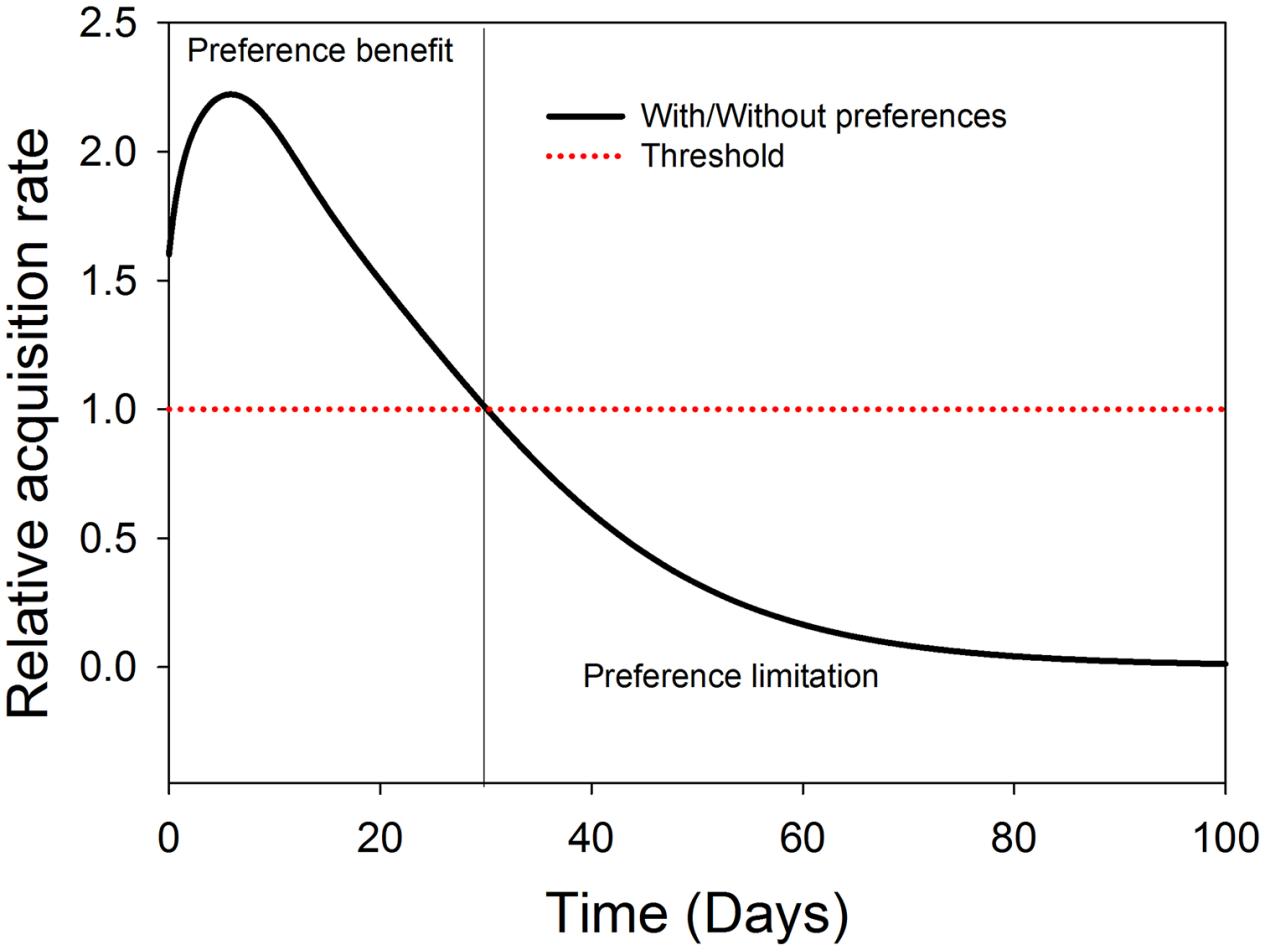
Relative acquisition rate of TSWV by *F*. *occidentalis* in the presence of TSWV induced preferential behaviour. The dotted line represents the threshold indicating when *F*. *occidentalis* preferences has no effect on the TSWV acquisition rate, and the vertical line represents the time at which the threshold is reached.

**Fig 3:**
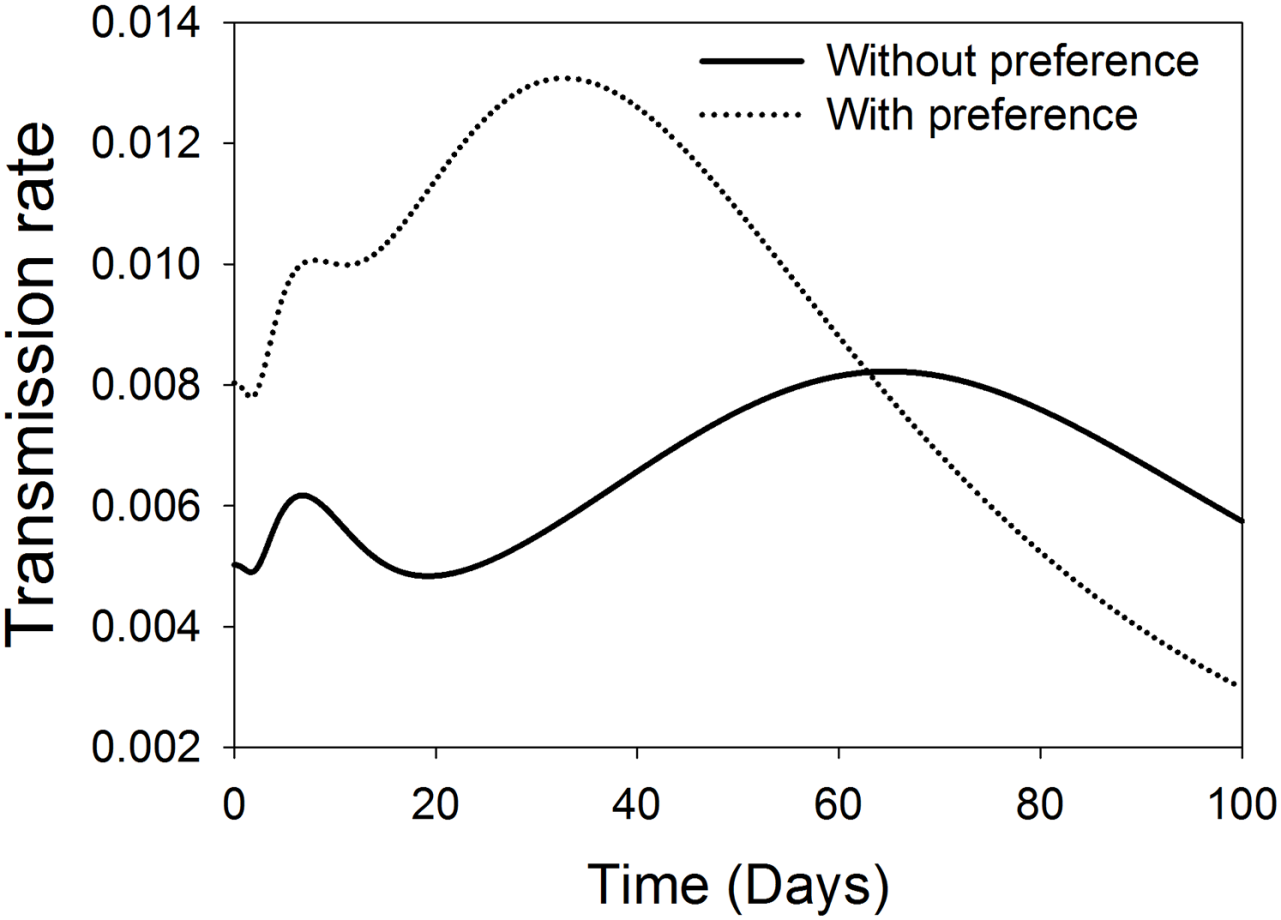
Comparison of effect of TSWV induced preferential behaviour on the transmission rate of *F*. *occidentalis*. The solid and the dotted lines represent the dynamics of the transmission rate of TSWV to host plants in the absence and in the presence of preference effects respectively.

In terms of TSWV spread dynamics, *F*. *occidentalis* relative acquisition rate and virus transmission rate to healthy host plants were modelled in the presence and absence of virus induced preferential behaviours of the vector. The results of the relative acquisition rate show that the preferential behaviour effect was beneficial above the threshold, and only up to the first 30 days after TSWV introduction into a crop stand. Thereafter, the influence of preference on acquisition is limited (Fig 2). On the other hand, the rate of TSWV transmission by *F*. *occidentalis* is greatly influenced by the preferential behaviour of the vector. The highest influence of preference on the rate of transmission being in the first approximately 63 days after TSWV introduction into a crop stand. Thereafter, the influence of preference is limited due to the reducing fraction of the healthy plants (Fig 3).

#### 2. Host plants abundance dynamics

**Fig 4:**
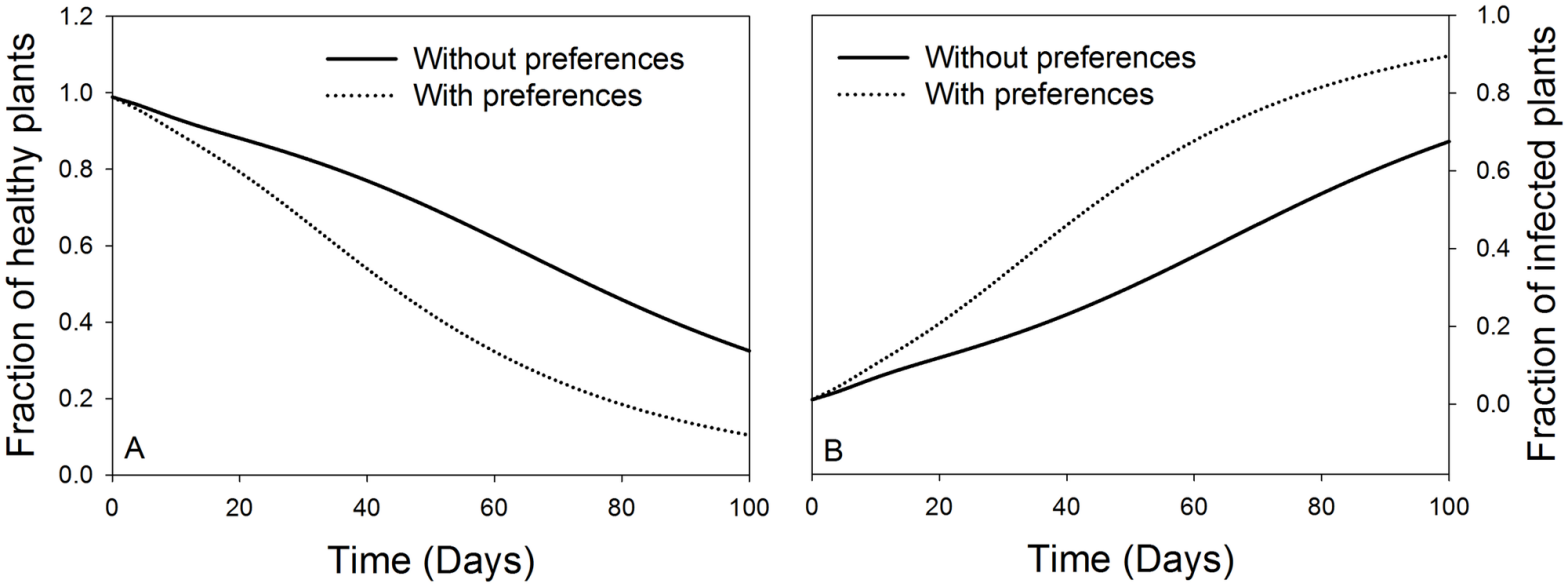
Influence of virus induced preferential behaviour of *F*. *occidentalis* on host plant abundance. A. Represent fraction of healthy plants overtime. B. Represent fraction of infected plants overtime.

It can be observed that the influence of the virus induced vectors’ preference in the first 100 days of TSWV introduction in a crop stand accelerates the transmission of the disease to healthy plants. The model estimates that preferences of viruliferous vectors (infected and transmitters) to healthy plants may increase the transmission rate by up to 33.3%. The consequence can lead to up to 30% decrease in the fraction of healthy plants (Fig 4A). On the other hand, the influence of *F*. *occidentalis* preferential behaviour on the fraction of the TSWV infected host plants over time indicates a steady rise of the infected plants. Thus, the TSWV induced preferential behaviour benefits the virus in terms of spread (Fig 4B).

#### 3. Vector abundance dynamics model—Adults

**Fig 5:**
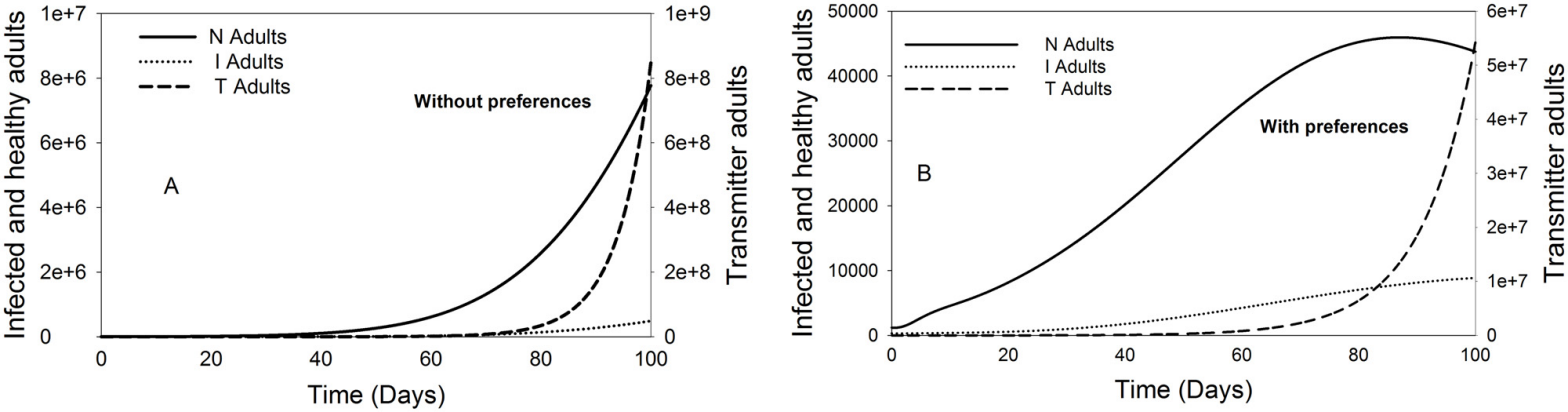
Population dynamics of adult *F*. *occidentalis* in the presence and absence of preference effects. A. Represents the adult population dynamics without the influence of TSWV induced preferential behaviour. B. Represents the adult population dynamics with the influence of TSWV induced preferential behaviour. The initials N, I, and T, refer to healthy, infected and transmitters respectively.

**Fig 6:**
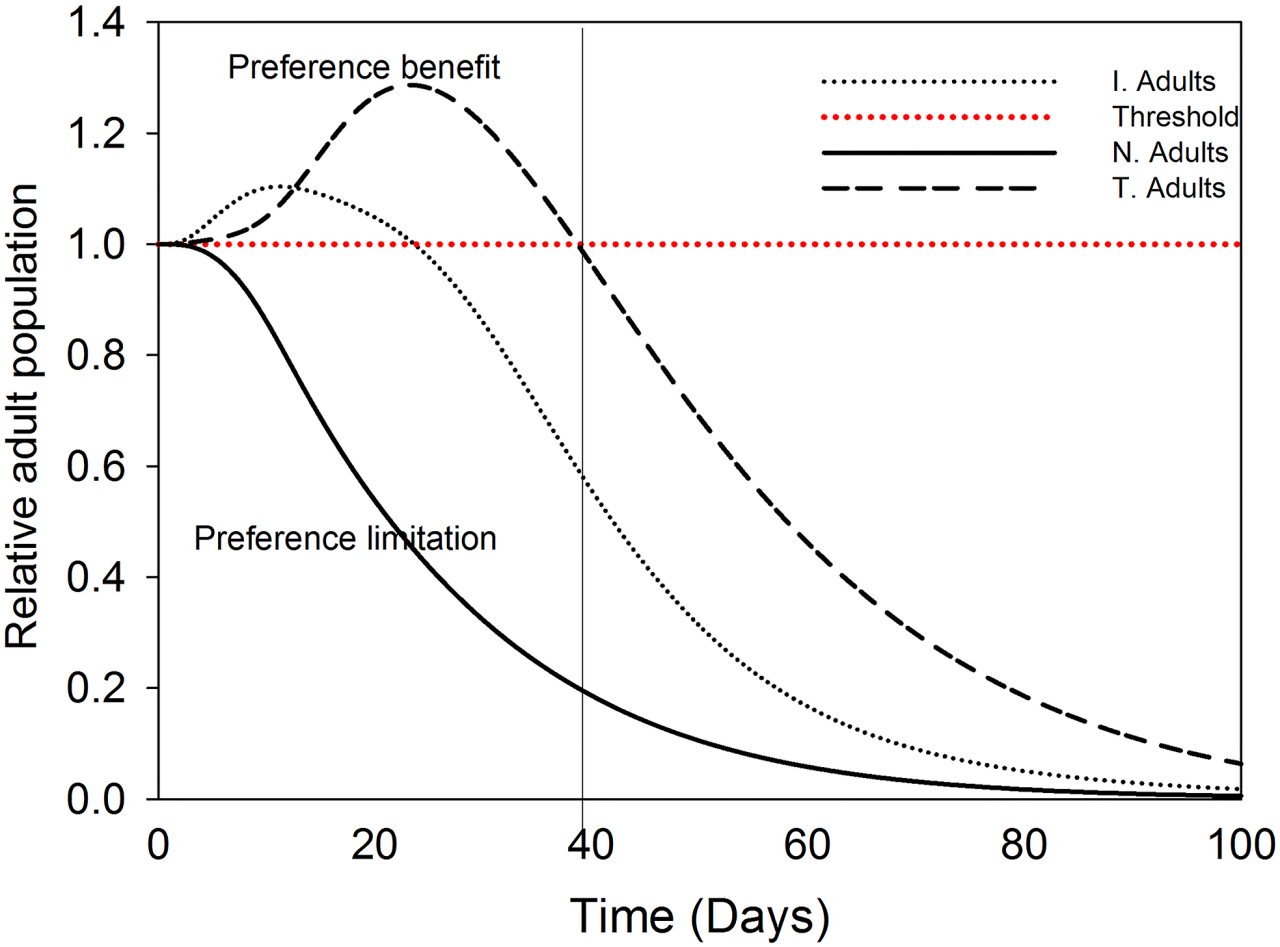
Relative population dynamics of *F*. *occidentalis* adults in the presence and absence of preference effects. Population dynamics of *F*. *occidentalis* adults’ in the presence relative to the absence of TSWV induced preferential behaviour. The initials N, I, and T, refer to healthy, infected and transmitters respectively.

Since adult is the crucial stage for TSWV spread due to its mobility and host plant choice behaviour, it was the only stage in *F*. *occidentalis*’ life cycle considered; in terms of vector abundance, for the analysis of the influence of TSWV induced preferential behaviour in the first 100 days of the virus introduction in a crop population. This was based on our listed assumptions. In the absence of preference, the population of the transmitter adults shows exponential rise compared to the healthy and the infected adults (Fig 5A). By introducing TSWV induced preferential behaviour to the model, the transmitters population increases exponentially 80 days after the virus introduction in the crop stand (Fig 5B). A comparison of the adults’ abundance ratios shows clearly that the benefit of virus induced vector preference is only available within the first 22 and 40 days after the introduction of TSWV in the crop stand, only for the infected and transmitter adults respectively, but not for the healthy adults’ population, after these days the influence of preference is limited (Fig 6).

### B. TSWV induced effects on *F*. *occidentalis* life expectancy in TSWV-vector-host plant interaction dynamics

The influence of TSWV on *F*. *occidentalis* development time and survival was modelled in terms of adults’ life expectancy, and its contribution to the different aspects of disease development.

#### 1. Disease spread dynamics (Acquisition/ Transmission)

**Fig 7:**
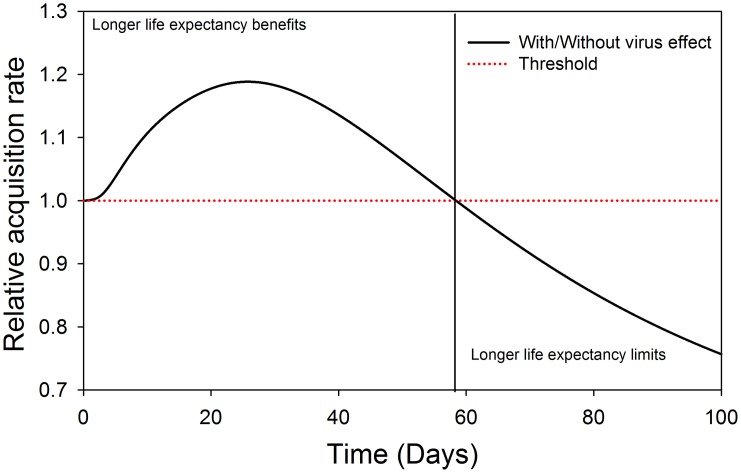
Relative acquisition rate dynamics of *F*. *occidentalis*, when TSWV has an effect with respect to no effect on *F*. *occidentalis* life expectancy. The dotted line represents the threshold; indicating when TSWV effect on *F*. *occidentalis* life expectancy does not influence the acquisition rate, and the vertical line represents the time at which the threshold is reached.

**Fig 8:**
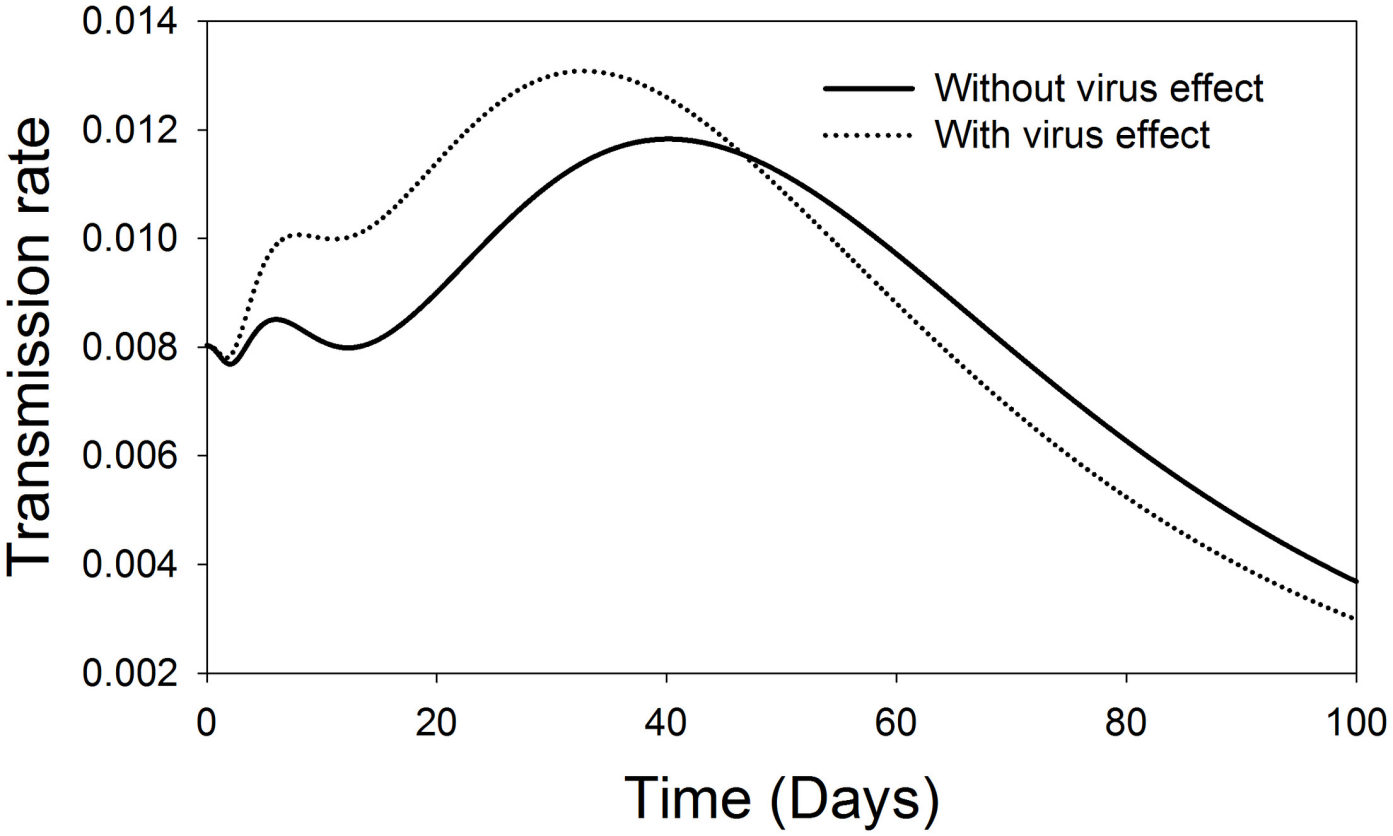
Comparison of the TSWV induced effect on *F*. *occidentalis* life expectancy on the transmission rate of TSWV. The solid line represents the transmission rate of TSWV to plants in the absence of TSWV induced effect on life expectancy over time, and the dotted line represents transmission rate in the presence of TSWV induced effect on life expectancy.

Looking at the acquisition ratio of TSWV with respect to life expectancy of the adult *F*. *occidentalis* in the presence or absence of virus effects, the benefits of the virus influences are observed above the threshold and only in the first 58 days after TSWV introduction into a crop population. Thereafter the influence is limited (Fig 7). For the transmission rate to healthy plants in the presence of TSWV effects, the benefits of *F*. *occidentalis* improved life expectancy are observed only in the first 46 days after virus introduction, there after the effects are limited. The opposite applies for situation without virus effects (Fig 8).

#### 2. Host plants abundance dynamics

**Fig 9:**
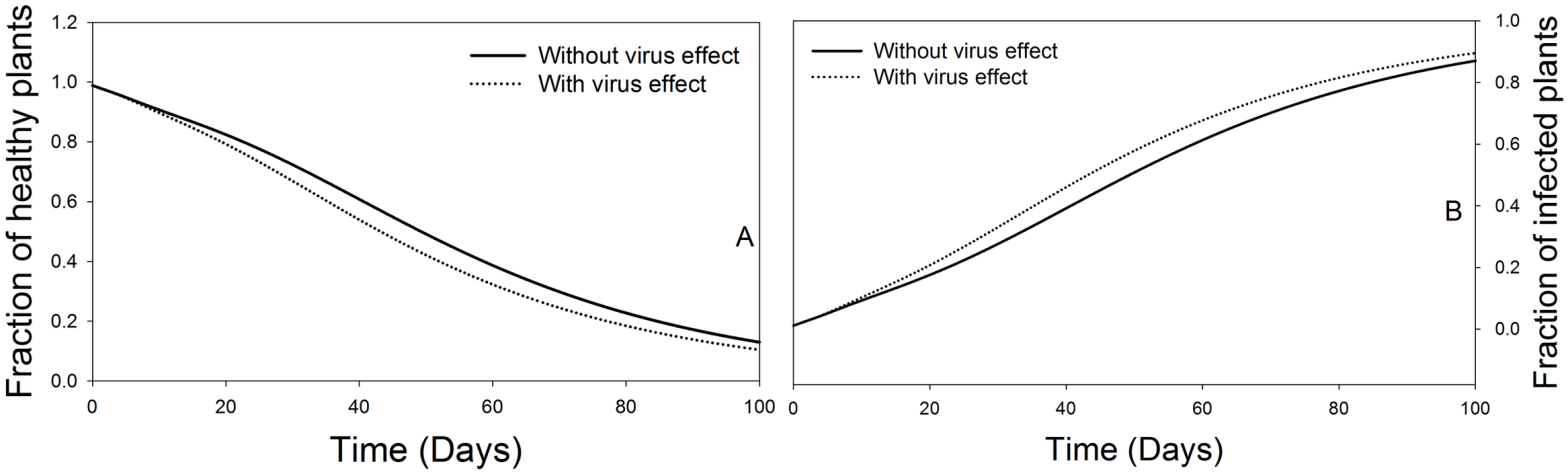
Influence of TSWV induced effect on *F*. *occidentalis* life expectancy on host plants abundance over time. A. Represents the fraction of healthy plants, and B. Represents the fraction of infected plants. Dotted and solid lines represent the dynamics of the fraction of plants, ‘with’ and ‘without’ TSWV induced effects on *F*. *occidentalis* life expectancy respectively.

The influence of life expectancy of TSWV infected *F*. *occidentalis* on host plants abundance both in the presence or absence of TSWV effects results in a steady decline in the fraction of healthy host plants in a given population, with the presence of TSWV effect resulting in slightly lower fractions of healthy plants compared to the absence of TSWV effects (Fig 9A). While for the infected host plants, there is a steady rise, for both with or without TSWV effects. However, inclusion of the virus effects in the model slightly increases the fraction of infected host plants compared to neglecting it (Fig 9B).

#### 3. Vector abundance dynamics model—Adults

**Fig 10:**
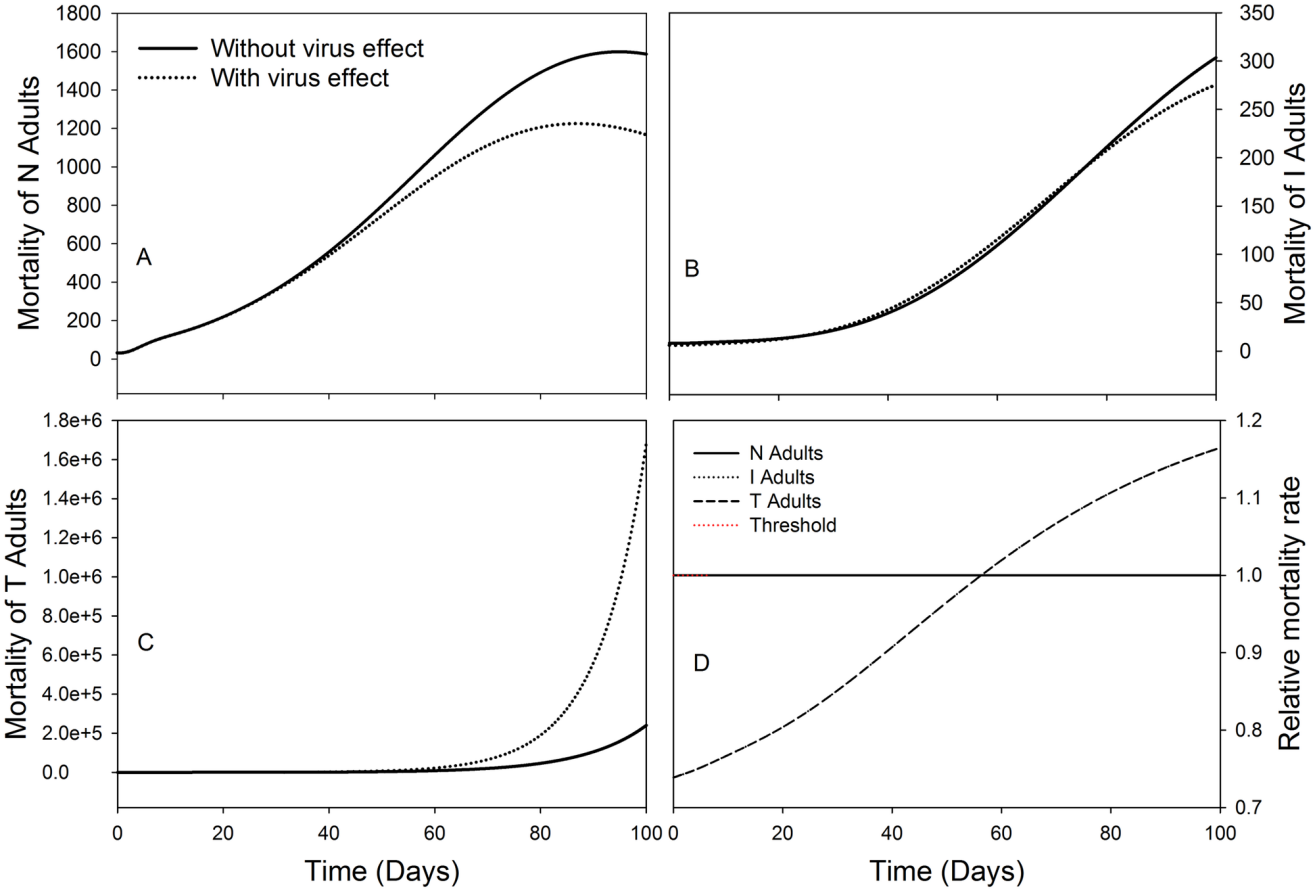
Dynamics of adults’ life expectancy as influenced by ‘with’ or ‘without’ virus effects on *F*. *occidentalis* mortality over time. A. On healthy adults, B. On infected adults, C. On transmitter adults and D. Represent the relative mortality rate over time for each adult cohort under the TSWV induced effect on *F*. *occidentalis* life expectancy. The initials N, I, and T, refer to healthy, infected and transmitters respectively.

Regarding the population dynamics of the three vector cohorts, the influence of life expectancy was modelled based on adults’ mortality in each cohort, both with and without virus effects (Fig 10). For the healthy adults (Fig 10A), an influence of the presence of virus effects on life expectancy with respect to mortality is observed 40 days after virus introduction in a crop stand, resulting in reduced mortality compared to the absence of virus effects. For the infected adults (Fig 10B), the influence is minimal with a small difference being observed 80 days after TSWV introduction. While for the transmitter adults (Fig 10C), the influence is observed 60 days after virus introduction, with significantly higher mortality in the presence of virus effects compared to without virus effects.

When modelling the virus influences on life expectancy with respect to the relative mortality rate (Fig 10D), for both infected and transmitter adults’ cohorts, the rate increases steadily at the same pace in their respective populations. However, for the non-infected adults there is no influence on the rate of mortality, as the only expected influence would be in the presence of virus effects, but since the status of the non-infected adults change to infected adults when they come in contact with the virus, the virus effects do not affect the healthy population in terms of the relative mortality rate, and thus equals to the threshold.

#### 4. Relative adults population dynamics model

**Fig 11:**
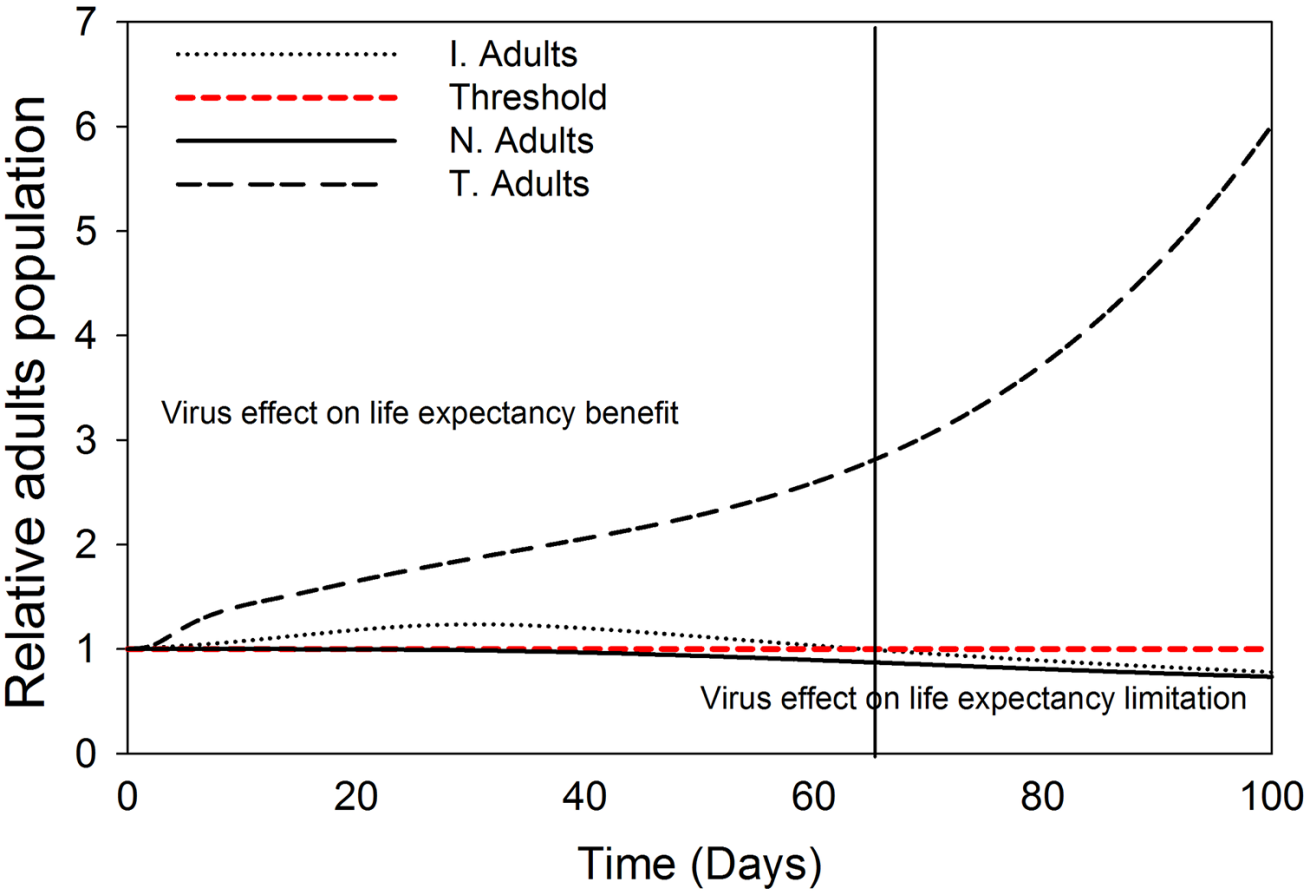
Relative *F*. *occidentalis* adult population; ‘with’ with respect to ‘without’ TSWV effect, on *F*. *occidentalis* life expectancy. The red dashed line represents the threshold indicating when TSWV effect on *F*. *occidentalis* life expectancy does not influence the adult population and the vertical line represents the time at which the threshold is reached. The initials N, I, and T, refer to healthy, infected and transmitters respectively.

Evaluation of the relative adults’ population dynamics (with/without virus effects on life expectancy) of different vector cohorts in the first 100 days after TSWV introduction in a crop population shows a positive virus effect on the dynamics of transmitter adults’ population with a steady rise over time observed above the threshold. For the infected adults’ population, there is also a positive effect of the virus, however the rise is minimal, only slightly above the threshold, and the effects are limited to 60 days after virus introduction (Fig 11). For the healthy adults’ population, there are no virus effects observed in the first 40 days after virus introduction, but thereafter, the effects are negative.

## Discussion

Since TSWV transmission is dependent on the vector biology and behaviour, both direct and indirect host plant mediated virus effects on the vector are crucial for the prediction of the virus epidemics. The hereby reported modelling of disease spread dynamics which involves the virus acquisition and transmission rate by the vector, is linked to the observed dynamics of the host plants and vectors abundance in relation to the vectors’ life expectancy and preferential behaviour as influenced by the virus. With the introduction of TSWV in a crop population, the fraction of the healthy plants is expected to diminish over time due to the progressive transmission of the virus; this means a change in the host plant status from healthy to infected, and thus, an increase in the ratio of infected plants. Considering the reported TSWV induced preferential behaviour of *F*. *occidentalis* [29][42][57], the non-infected adults tend to choose the infected plants over the healthy plants both for feeding and egg laying, leading to more eggs laid in the infected plants resulting in a population outburst of transmitter adults in the following generation, and thus, further TSWV spread to the next available healthy plant. This explains the high populations of the transmitter adults observed over time after the introduction of the virus. The observed steady increase in the fraction of the infected host plants is expected to reach a plateau after a certain time period (beyond the 100 days), this is because the fraction of the healthy host plants is decreasing at the same rate, and therefore the influence of preference is limited, which also limits further transmission. Such vector-modified behaviour as a result of virus influences is a common trait among the *Tospoviruses*. This phenomenon has also been reported in other virus-vector systems, for example the cereal aphids *Rhadopalosiphum padi* and *Myzus persicae*, after acquisition of *Barley yellow dwarf virus* (BYDV) and *Potato leafroll virus* (PLRV) respectively [43][58][59][60][61][62], and also in whiteflies *Bemisia tabaci* after acquisition of *Tomato yellow leaf curl virus* (TYLCV) [63]. Several other factors; besides virus manipulation, have also been reported to contribute to the observed behavioural changes on the vector. For instance, differences in colour and odour between infected and non-infected host plant have been linked to this behaviour. Aphids and thrips are reported to be more attracted to host plants infected with virus due to colour change (yellowing) and odour cues [64][23][24]. However, in all these reports the insects had no prior exposure to the virus and were considered healthy, which represents only one path of our model where virus free adults that developed on healthy plant are attracted to the infected host plants. For the other two paths of the model; the infected and the transmitter adults, colour and odour cues do not seem to play any role, therefore, the only explanation would be the manipulation of the vector by the virus to enhance its transmission and spread, a mechanism which could be linked to an evolutionary interaction between plant viruses and their vectors [43].

The observed increase in life expectancy of *F*. *occidentalis* in response to TSWV also showed some influences on the disease spread dynamics. In the relative acquisition and the transmission rates of the vector, the virus effects on life expectancy are obviously positive primarily in the early stages of the disease development, but thereafter tending towards negative. This relates back to the TSWV induced preferential behaviour [24][42], and its dependent on the health status of the host plant. In the adults’ abundance model, the mortality of the healthy adults follows a normal sigmoid function with or without virus effects. The observed slight reduction with virus effect could be attributed to the reduced population of healthy individuals due to adults switching status, i.e. from healthy to infected, as a result of contact (acquisition) with a virus infected host plant, and is again a consequence of the virus induced preferential behaviour[24][42]. For the infected adults, the mortality is expected to increase steadily with or without virus effects. However, after 80 days there is a slight reduction in mortality due to virus effects: as the virus induced preferential behaviour manipulates the infected adults to choose healthy host plants, thus reducing mortality. The transmitter adults experience an exponential rise in mortality with virus effects after 60 days of disease progression over time. This is because the fraction of the healthy plants is reducing due to the increasing rate of transmission as a result of the rising numbers of the transmitter adults, leading to an increase in the fraction of the infected plants, and thus an equal increase in mortality due to limited preferential effects. The increase in life expectancy for the viruliferous paths compared to the healthy path of the vector as displayed in this model, is consequently dependent on the virus induced preferential behaviour of the vector, and the fraction of the healthy host plants [42]. The increased fitness of *F*. *occidentalis* exposed to TSWV has been associated with triggered immunity [48], as the insect lack an adaptive immune system [49]. This explains the observed increase in survival of the viruliferous populations on healthy host plants [24].

A phenomenon termed “Vector Manipulation Hypothesis” by Ingwell et al. [43], was the motivation of our study. It describes an evolutionary mechanism in plant pathogen–vector interactions that promote the multiplication of the pathogen and transmission to new host plants. Therefore, incorporating virus manipulated vector’s life processes in the predictive models would solve the problem of over or under estimation, as it is evident that this aspect is important for a realistic description of disease incidences by predictive models [38]. A more wholistic expansion of our model would be incorporation into a detailed weather based model, as well as inclusion of various aspects in the *Tospovirus*-vector-hostplant interaction like; vector gender, individual vector genetics, among others.

In conclusion, the dynamics of the complex and specific TSWV-*F*. *occidentalis*-host plant interaction, with focus on virus induced and host plant-mediated effects on the vector’s life processes, is a new contribution to the currency of the predictive models. A thorough understanding of the vector’s life processes as influenced by the virus, and their contribution to the virus-vector-hostplant interaction, is critical for a more precise prediction of TSWV epidemics.

## References

[1] Whitfield AE, Ullman DE, German TL. Tospovirus-Thrips Interactions. Annu Rev Phytopathol. 2005;43: 459–489. 10.1146/annurev.phyto.43.040204.140017 16078892

[2] van de Wetering F, van der Hoek M, Goldbach R, Peters D. Differences in tomato spotted wilt virus vector competency between males and females of Frankliniella occidentalis. Entomol Exp Appl. 1999;93: 105–112. 10.1046/j.1570-7458.1999.00567.x

[3] Wijkamp I, Almarza N, Goldbach R, Peters D. Distinct Levels of Specificity in Thrips Transmission of Tospoviruses. Phytopathology. 1995 p. 1069 10.1094/Phyto-85-1069

[4] Nagata J, Peters D. An anatomical perspective of tospovirus transmission. Virus-Insect-Plant Interact. 2001; 51–67.

[5] Scholthof KBG, Adkins S, Czosnek H, Palukaitis P, Jacquot E, Hohn T, et al Top 10 plant viruses in molecular plant pathology. Mol Plant Pathol. 2011;12: 938–954. 10.1111/j.1364-3703.2011.00752.x 22017770 PMC6640423

[6] Sherwood J, German T, Moyer J, Ullman D, Whitfield A. Tomato spotted wilt In: Maloy OC MT, editor. Encyclopedia of Plant Pathology. (ed by O.C. New York: John Wiley & Sons; 2000 pp. 1030–1031.

[7] Nagata T, Inoue-Nagata AK, Smid HM, Goldbach R, Peters D. Tissue tropism related to vector competence of Frankliniella occidentalis for tomato spotted wilt tospovirus. J Gen Virol. 1999;80 (Pt 2): 507–15. Available: http://www.ncbi.nlm.nih.gov/pubmed/10073714 10073714 10.1099/0022-1317-80-2-507

[8] Chatzivassiliou EK, Peters D, Katis NI. The Efficiency by Which Thrips tabaci Populations Transmit Tomato spotted wilt virus Depends on Their Host Preference and Reproductive Strategy. Phytopathology. 2002;92: 603–9. 10.1094/PHYTO.2002.92.6.603 18944256

[9] Ullman D, Cho J, Mau R, Westcot D, DM C, Custer D. Midgut epithelial cells act as a barrier to Tomato spotted wilt virus acquisition by adult western flower thrips. Phytopathology. 1992;82: 1333–1342.

[10] Wijkamp I, Peters D. Determination of Median Latent Period of two Tospoviruses in WFT, using novel leaf disk assay. Phytopathology. 1993;83: 986–991.

[11] van de Wetering F, Goldbach R, Peters D. Tomato Spotted Wilt Tospovirus Ingestion by First Instar Larvae of Frankliniella occidentalis is a Prerequisite for Transmission. Phytopathology. 1996 pp. 900–905.

[12] Wijkamp I, van Lent J, Kormelink R, Goldbach R, Peters D. Multiplication of tomato spotted wilt virus in its insect vector, Frankliniella occidentalis. J Gen Virol. 1993;74 (Pt 3): 341–9. 10.1099/0022-1317-74-3-341 8445364

[13] Ullman D, German T, Sherwood J. Tospovirus replication in insect vector cells: Immunocytochemical evidence that the nonstructural protein encoded by the S RNA of tomato spotted wilt [Internet]. Phytopathology. 1993 Available: http://www.apsnet.org/publications/phytopathology/backissues/Documents/1993Articles/Phyto83n04_456.PDF

[14] Nagata T, Inoue-Nagata AK, van Lent J, Goldbach R, Peters D. Factors determining vector competence and specificity for transmission of Tomato spotted wilt virus. J Gen Virol. 2002;83: 663–71. Available: http://www.ncbi.nlm.nih.gov/pubmed/11842261 11842261 10.1099/0022-1317-83-3-663

[15] Moritz G, Kumm S, Mound L. Tospovirus transmission depends on thrips ontogeny. Virus Res. 2004;100: 143–9. 10.1016/j.virusres.2003.12.022 15036845

[16] Hogenhout SA, Ammar E-D, Whitfield AE, Redinbaugh MG. Insect vector interactions with persistently transmitted viruses. Annu Rev Phytopathol. 2008;46: 327–59. 10.1146/annurev.phyto.022508.092135 18680428

[17] Ullman D, German T, Sherwood J, Westcot D. Thrips transmission of tospoviruses: future possibilities for management In: Herausgeber: Parker, Bruce L., Skinner, Margaret, Lewis T, editor. Thrips Biology and Management. Springer US; 1995 pp. 135–151. 10.1007/978-1-4899-1409-5_19

[18] Ullman D, Sherwood J, German T. Thrips as a vector of plant pathogens. In Thrips as Crop Pests’'. In: Lewis T, editor. Thrips as Crop Pests. Wallingford, U.K: CAB International,; 1997.

[19] de Assis Filho FM, Naidu RA, Deom CM, Sherwood JL. Dynamics of Tomato spotted wilt virus Replication in the Alimentary Canal of Two Thrips Species. Phytopathology. 2002;92: 729–33. 10.1094/PHYTO.2002.92.7.729 18943268

[20] de Assis Filho FM, Deom CM, Sherwood JL. Acquisition of Tomato spotted wilt virus by Adults of Two Thrips Species. Phytopathology. 2004;94: 333–336. 10.1094/PHYTO.2004.94.4.333 18944108

[21] Stafford-Banks C, Rotenberg D, Johnson BR, Whitfield AE, Ullman DE. Analysis of the salivary gland transcriptome of Frankliniella occidentalis. PLoS One. 2014;9: e94447 10.1371/journal.pone.0094447 24736614 PMC3988053

[22] Stumpf CF, Kennedy GG. Effects of tomato spotted wilt virus isolates, host plants, and temperature on survival, size, and development time of Frankliniella occidentalis. Entomol Exp Appl. 2007;123: 139–147. 10.1111/j.1570-7458.2007.00541.x

[23] Maris PC, Joosten NN, Goldbach RW, Peters D. Tomato spotted wilt virus Infection Improves Host Suitability for Its Vector Frankliniella occidentalis. Phytopathology. 2004;94: 706–11. 10.1094/PHYTO.2004.94.7.706 18943902

[24] Ogada PA, Maiss E, Poehling H-M. Influence of tomato spotted wilt virus on performance and behaviour of western flower thrips (Frankliniella occidentalis). J Appl Entomol. 2013;137: 488–498.

[25] Shrestha A, Srinivasan R, Riley DG, Culbreath AK. Direct and indirect effects of a thrips-transmitted Tospovirus on the preference and fitness of its vector, Frankliniella fusca. Entomol Exp Appl. 2012;145: 260–271. 10.1111/eea.12011

[26] Robb K. Analysis of Franklineilla occidentalis (Pergande) as a pest of floricultural crips in California greenhouses. University of Califonia, Riverside 1989 135

[27] Sakimura K. Frankliniella fusca, an additional vector for the tomato spotted wilt virus, with notes on Thrips tabaci, another vector. Phytopathology. 1963;53: 412–415.

[28] Wijkamp I, Goldbach R, Peters D. Propagation of tomato spotted wilt virus in Frankliniella occidentalis does neither result in pathological effects nor in transovarial passage of the virus. 1996; 285–292.

[29] Stafford CA, Walker GP, Ullman DE. Infection with a plant virus modifies vector feeding behavior. Proc Natl Acad Sci U S A. 2011;108: 9350–9355. 10.1073/pnas.1100773108 21606372 PMC3111272

[30] Jones RAC, Salam MU, Maling TJ, Diggle AJ, Thackray DJ. Principles of Predicting Plant Virus Disease Epidemics. Annu Rev Phytopathol. 2010;48: 179–203. 10.1146/annurev-phyto-073009-114444 20433348

[31] Olatinwo RO, Paz JO, Brown SL, Kemerait RC, Culbreath a K, Beasley JP, et al A predictive model for spotted wilt epidemics in peanut based on local weather conditions and the tomato spotted wilt virus risk index. Phytopathology. 2008;98: 1066–74. 10.1094/PHYTO-98-10-1066 18943452

[32] Yudin LS, Tabashnik BE, Cho JJ, Mitchell WC. Disease-prediction and economic models for managing tomato spotted wilt virus disease in lettuce. Plant Dis. 1990;74: 211–216.

[33] De Wolf ED, Isard SA. Disease cycle approach to plant disease prediction. Annu Rev Phytopathol. 2007;45: 203–220. 10.1146/annurev.phyto.44.070505.143329 17408356

[34] Magarey RD, Fowler GA, Borchert DM, Sutton TB, Colunga-Garcia M, Simpson JA. NAPPFAST: An Internet System for the Weather-Based Mapping of Plant Pathogens. Plant Dis. 2007;91: 336–345. 10.1094/PDIS-91-4-0336 30781172

[35] Morsello SC, Kennedy GG. Spring temperature and precipitation affect tobacco thrips, Frankliniella fusca, population growth and Tomato spotted wilt virus spread within patches of the winter annual weed Stellaria media. Entomol Exp Appl. 2009;130: 138–148. 10.1111/j.1570-7458.2008.00801.x

[36] Morsello SC, Beaudoin ALP, Groves RL, Nault BA, Kennedy GG. The influence of temperature and precipitation on spring dispersal of Frankliniella fusca changes as the season progresses. Entomol Exp Appl. 2010;134: 260–271. 10.1111/j.1570-7458.2009.00959.x

[37] Madden L, Raccah B, Pirone T. Modeling plant disease increase as a function of vector numbers: non persistent viruses. Res Popul Ecol (Kyoto). 1990;32: 47–65.

[38] Chappell TM, Beaudoin ALP, Kennedy GG. Interacting virus abundance and transmission intensity underlie tomato spotted wilt virus incidence: an example weather-based model for cultivated tobacco. PLoS One. 2013;8: e73321 10.1371/journal.pone.0073321 23977384 PMC3747132

[39] Madden L, Knoke J, Louie R. The statistical relationship between aphid trap catches and maize dwarf mosaic virus inoculation pressure In: Plumb R, Thresh J, editors. Plant Virus Epidemiology. Oxford: Blackwell Publishing Ltd; 1983 pp. 159–168.

[40] Jeger MJ, Madden LV, Bosch F. The effect of transmission route on plant virus epidemic development and disease control. J Theor Biol. 2009;258: 198–207. 10.1016/j.jtbi.2009.01.012 19490879

[41] Jeger M, van den Bosch F, McRoberts N. Modelling transmission characteristics and epidemic development of the tospovirus—thrip interaction. Arthropod-Plant Interactios. 2015; 107–120. 10.1007/s11829-015-9363-2

[42] Shalileh S, Ogada PA, Poehling HM. Manipulation of Frankliniella occidentalis by tomato spotted wilt virus (Tospovirus) via the host plant nutrients to enhance transmission and spread of the virus. Environ Entomol. 2016; in press. 10.1093/ee/nvw102 PMC5037971 27566527

[43] Ingwell LL, Eigenbrode SD, Bosque-Pérez NA. Plant viruses alter insect behavior to enhance their spread. Sci Rep. 2012;2: 578 10.1038/srep00578 22896811 PMC3419366

[44] Thomas F, Adamo S, Moore J. Parasitic manipulation: where are we and where should we go? Behav Processes. 2005;68: 185–199. 10.1016/j.beproc.2004.06.010 15792688

[45] Carmo-sousa M, Moreno A, Garzo E, Fereres A. A non-persistently transmitted-virus induces a pull—push strategy in its aphid vector to optimize transmission and spread. Virus Res. Elsevier B.V.; 2014;186: 38–46. 10.1016/j.virusres.2013.12.012 24373951

[46] Rajabaskar D, Bosque-pérez NA, Eigenbrode SD. Preference by a virus vector for infected plants is reversed after virus acquisition. Virus Res. Elsevier B.V.; 2014;186: 32–37. 10.1016/j.virusres.2013.11.005 24269348

[47] Roosien BK, Gomulkiewicz R, Ingwell LL, Bosque-pe NA. Conditional Vector Preference Aids the Spread of Plant Pathogens: Results From a Model. Environ Entomol. 2013;42: 1299–1308. 10.1603/EN13062 24246613

[48] de Medeiros RB, Resende RDO, de Avila AC. The plant virus Tomato Spotted Wilt Tospovirus activates the immune system of its main insect vector, Frankliniella occidentalis. J Virol. 2004;78: 4976–82. 15113877 10.1128/JVI.78.10.4976-4982.2004 PMC400384

[49] Irving P, Troxler L, Heuer TS, Belvin M, Kopczynski C, Reichhart JM, et al A genome-wide analysis of immune responses in Drosophila. Proc Natl Acad Sci U S A. 2001;98: 15119–15124. 10.1073/pnas.261573998 11742098 PMC64993

[50] Jeger MJ, Van Den Bosch F, Madden L V, Holt J. A model for analysing plant-virus transmission characteristics and epidemic development. Ima J Math Appl Med Biol. 1998;15: 1–18. 10.1093/imammb/15.1.1

[51] Madden L V, Jeger MJ, van den Bosch F. A theoretical assessment of the effects of vector-virus transmission mechanism on plant virus disease epidemics. Phytopathology. 2000;90: 576–594. 10.1094/PHYTO.2000.90.6.576 18944537

[52] Ohnishi J, Knight LM, Hosokawa D, Fujisawa I, Tsuda S. Replication of Tomato spotted wilt virus After Ingestion by Adult Thrips setosus is Restricted to Midgut Epithelial Cells. Phytopathology. 2001;91: 1149–55. 10.1094/PHYTO.2001.91.12.1149 18943329

[53] Kritzman A, Gera A, Raccah B, van Lent JWM, Peters D. The route of tomato spotted wilt virus inside the thrips body in relation to transmission efficiency. Arch Virol. 2002;147: 2143–56. 10.1007/s00705-002-0871-x 12417949

[54] Naidu RA, Sherwood JL, Deom CM. Characterization of a vector-non-transmissible isolate of Tomato spotted wilt virus. Plant Pathol. 2008;57: 190–200. 10.1111/j.1365-3059.2007.01707.x

[55] Zitter TA, Daughtrey ML. Vegetable Crops: Tomato Spotted Wilt Virus. Available: [Internet]. 1989 p. Available: http://vegetablemdonline.ppath.cornell.edu/factshe.

[56] Robb KL, Parella MP, Newman JP. The biology and control of western flower thrips. Part I. Ohio Florosts Assoc Bull. 1988;699: 2–5.

[57] Bautista RC, Mau RFL, Cho JJ, Custer DM. Potential of Tomato Spotted Wilt Tospovirus Plant Hosts in Hawaii as Virus Reservoirs for Transmission by Frankliniella occidentalis (Thysanoptera: Thripidae).

[58] Medina-Ortega KJ, Bosque-Pérez NA, Ngumbi E, Jiménez-Martínez ES, Eigenbrode SD. Rhopalosiphum padi (Hemiptera: Aphididae) responses to volatile cues from Barley yellow dwarf virus-infected wheat. Environ Entomol. 2009;38: 836–845. 19508794 10.1603/022.038.0337

[59] Bosque-Pérez NA, Eigenbrode SD. The influence of virus-induced changes in plants on aphid vectors: insights from luteovirus pathosystems. Virus Res. 2011;159: 201–5. 10.1016/j.virusres.2011.04.020 21549769

[60] Werner BJ, Mowry TM, Bosque-Pérez NA, Ding H, Eigenbrode SD. Changes in green peach aphid responses to potato leafroll virus-induced volatiles emitted during disease progression. Environ Entomol. 2009;38: 1429–1438. 19825298 10.1603/022.038.0511

[61] Jiménez-Martínez ES, Bosque-Pérez NA, Berger PH, Zemetra RS, Ding H, Eigenbrode SD. Volatile Cues Influence the Response of *Rhopalosiphum padi* (Homoptera: Aphididae) to Barley Yellow Dwarf Virus–Infected Transgenic and Untransformed Wheat. Environ Entomol. 2004;33: 1207–1216.

[62] Eigenbrode SD, Ding H, Shiel P, Berger PH. Volatiles from potato plants infected with potato leafroll virus attract and arrest the virus vector, Myzus persicae (Homoptera: Aphididae). Proc R Soc London Ser B-Biological Sci. 2002;269: 455–460. 10.1098/rspb.2001.1909 PMC1690919 11886636

[63] Moreno-Delafuente A, Garzo E, Moreno A, Fereres A. A plant virus manipulates the behavior of its whitefly vector to enhance its transmission efficiency and spread. PLoS One. 2013;8: e61543 10.1371/journal.pone.0061543 23613872 PMC3629040

[64] Döring TF, Archetti M, Hardie J. Autumn leaves seen through herbivore eyes. Proc Biol Sci. 2009;276: 121–127. 10.1098/rspb.2008.0858 18782744 PMC2614250

